# Antimicrobial susceptibility patterns from urinary isolates obtained from cats (2013‐2020)

**DOI:** 10.1111/jvim.16711

**Published:** 2023-04-19

**Authors:** Caitlan W. Koontz, Steven E. Epstein, Jodi L. Westropp

**Affiliations:** ^1^ William R. Pritchard Veterinary Medical Teaching Hospital, School of Veterinary Medicine University of California, Davis Davis California 95616 USA; ^2^ Department of Veterinary Surgical and Radiological Sciences, School of Veterinary Medicine University of California, Davis Davis California 95616 USA; ^3^ Department of Veterinary Medicine and Epidemiology, School of Veterinary Medicine University of California, Davis Davis California 956161 USA

**Keywords:** kidney, multidrug resistance, pyelonephritis, renal, subclinical bacteriuria, urinary tract, urinary tract infections

## Abstract

**Background:**

Bacterial urinary tract infections have been associated with comorbidities and increased antimicrobial resistance over time.

**Objective:**

To identify bacterial species, antimicrobial susceptibility patterns and risk factors associated with antimicrobial resistance.

**Animals:**

Three hundred sixty‐three positive urine cultures from 308 cats.

**Methods:**

Bacterial species and antimicrobial susceptibility data from positive aerobic bacterial urine cultures from cats with growth of ≥10^3^ colony forming units per milliliter (cfu/ml) were included. Medical records were reviewed, and bacteriuria was classified as sporadic bacterial cystitis, recurrent bacterial cystitis or subclinical bacteriuria (SBU). Multivariable logistic regression analysis was used to evaluate antimicrobial resistance risk factors.

**Results:**

A total of 444 bacterial isolates from 363 bacteriuric episodes were identified. *Escherichia coli* (52%) and SBU (59%) were the most common organism and classification, respectively. When compared to other classifications of bacteriuria, *Enterococcus* spp. were more likely to be isolated from SBU episodes (*P* < .001), whereas *E. coli* was more likely to be isolated from sporadic bacterial cystitis episodes (*P* < .001). Recurrent bacterial cystitis was associated with an increased risk of antimicrobial resistance to amoxicillin/clavulanic acid (odds ratio [OR], 3.9; 95% confidence interval [CI], 1.3‐11.3). The percent susceptibilities of all bacterial isolates to commonly prescribed antimicrobials were amoxicillin/clavulanic acid (72%), cefazolin (49%), enrofloxacin (61%), and trimethoprim/sulfamethoxazole (75%). Multidrug resistance was highest for *Enterococcus faecium* isolates (65%).

**Conclusions and Clinical Importance:**

No antimicrobial achieved >90% susceptible designation to all bacteria isolated highlighting the importance of performing urine culture and susceptibility testing, particularly for cats with recurrent bacterial cystitis.

**Abbreviations**
ABUC: aerobic bacterial urine culture
CFU: colony forming units
CI: confidence interval
CLSI: Clinical and Laboratory Standards Institutes
ISCAID: International Society for Companion Animal Infectious Diseases
LUTS: lower urinary tract signs
MDR: multidrug resistant
OR: odds ratio
SBU: subclinical bacteriuria
UTI: urinary tract infection

## 1 INTRODUCTION

The reported prevalence of urinary tract infections (UTI) in cats has varied considerably over the past 30 years. Previous publications from secondary or tertiary referral veterinary hospitals reported that <4% of cats with lower urinary tract signs (LUTS) had positive urine culture results.^1^, ^2^ Several studies from Europe, 1 from Thailand and 1 from the United States reported that 4%‐33% of cats were diagnosed with UTI, but clinical signs were not always reported. Some of these cats might have had subclinical bacteriuria (SBU), which often is diagnosed as an incidental finding.^3^, ^4^, ^5^, ^6^, ^7^, ^8^, ^9^, ^10^ The signalment and comorbidities present in cats across these studies also varied greatly. Comorbidities such as diabetes mellitus, kidney disease and micturition disorders have been reported in cats with bacterial cystitis and SBU.^11^, ^12^, ^13^, ^14^, ^15^, ^16^ Bacterial cystitis and SBU also have been reported to occur more often in older female cats, but control groups to confirm this finding were often missing.^2^, ^11^, ^14^, ^17^, ^18^, ^19^ The presence of these predisposing factors may explain the diversity in the observed results, but regional differences also may contribute.

The term UTI implies the cat has clinical signs associated with positive aerobic bacterial urine culture (ABUC) and can be classified as sporadic or recurrent bacterial cystitis. Historically, UTIs were classified as simple uncomplicated or complicated,^20^ but use of this terminology is discouraged because not all cats with UTIs are screened for comorbidities and documentation of a comorbidity does not necessarily imply that an infection is complicated. In addition, it often does not alter treatment protocols. Current guidelines describe cats presenting with <3 UTIs in a 12‐month period as having sporadic bacterial cystitis and those with ≥3 as having recurrent bacterial cystitis.^21^ Recurrent bacterial cystitis can be further classified as relapsing or reinfection. Bacteriuria in the absence of clinical signs, known as SBU, accounts for a proportion of positive urine cultures in cats and often is discovered incidentally.^17^

*Escherichia coli* is the most commonly isolated organism from urine specimens in cats with UTIs and SBU and in 1 study accounted for 42.3% of isolates.^22^, ^23^ Other organisms include *Enterococcus* spp., *Staphylococcus* spp., *Streptococcus* spp., *Proteus* spp. and other gram‐negative bacteria.^22^ Although the isolates cultured from urine specimens from cats generally have not changed over the years,^23^ it is unknown if the antimicrobial susceptibility of these isolates has changed. Antimicrobial resistance is an emerging problem in both cats and humans.^24^, ^25^, ^26^ This resistance appears to be a worldwide issue with an increase in resistance to all antimicrobials from bacterial urine isolates noted between 2000‐2009 and 2009‐2014 in Germany.^23^, ^27^ However, only *E. coli* had high rates of antimicrobial resistance to amoxicillin and β lactams from urine isolates from cats in Taiwan.^28^ Our objectives were to (a) identify the bacterial species isolated from urine specimens of cats with bacterial cystitis and SBU, (b) evaluate the prevalence of individual bacterial species during the time period, and (c) determine risk factors for antimicrobial resistance in cats with UTI.

## 2 MATERIALS AND METHODS

### 2.1 Data collection

All urine samples obtained from cats by cystocentesis, nephropyelocentesis, and SC ureteral bypass port aspirate that had positive quantitative ABUC results with bacterial counts ≥10^3^ cfu/mL and subsequent antimicrobial susceptibility testing at the William R. Pritchard Veterinary Medical Teaching Hospital between January 1, 2013 and December 30, 2020 were identified from an electronic database. Urine specimens from cats collected via urinary catheter, voiding or other non‐sterile sample techniques were excluded. Urine specimens with growth of non‐bacterial pathogens (e.g., fungi) were excluded. Repeat cultures from the same cat in the same calendar year were excluded from analysis. However, for each cat that appeared >1 time during the entire study period, all positive cultures recorded during initial data collection were used to determine classification of bacteriuria. All bacterial species isolated from urine cultures were recorded, as well the number of isolates obtained from the individual urine specimens. If a cat had recurrent bacteriuria documented in subsequent calendar years, their signalment and all other recorded data were documented again to correspond to the time the positive ABUC was obtained, which we defined as a “bacteriuric episode.” Each bacteriuric episode represents 1 positive ABUC from 1 cat, regardless of whether the ABUC was monomicrobial or polymicrobial. The corresponding medical records from all cats were reviewed and the signalment (including age, breed, sex, and neuter status) and any comorbidities documented at the time of the bacteriuric episodes were recorded. Recent (within 30 days) or current antimicrobial administration at the time of urine culture submission was recorded. The antimicrobial drug name, dosage, and duration was not recorded.

Bacteriuric episodes were classified as sporadic bacterial cystitis, recurrent bacterial cystitis or SBU. Bacterial cystitis (sporadic or recurrent [≥3 UTI in a 12‐month time period])^21^ was differentiated from SBU based on the presence of ≥1 of the following clinical signs reported by the client: pollakiuria, stranguria, dysuria or gross hematuria. Cats that had SBU, but also met the criteria for recurrent UTI were classified as both SBU and recurrent. Attending clinicians diagnosed pyelonephritis in cats based on a positive ABUC obtained by nephropyelocentesis or cystocentesis, supportive history and clinical signs, ultrasonographic examination and clinicopathologic data, or a combination of these.^21^ Bacteriuric episodes were further classified as persistent or relapsing if an infection did not clear (as documented by repeat ABUC) while the patient was receiving appropriate antimicrobial treatment guided by an initial ABUC, or these terms were used in the clinical diagnosis section of the medical record. For routine ABUC, 10 μL of urine was inoculated onto 5% defibrinated sheep blood and MacConkey agars and incubated at 35°C in room air with added 5% CO_2_. All bacteria were identified using matrix‐assisted desorption‐ionization time‐of‐flight mass spectrometry (MALDI‐TOF MS Biotyper, Bruker Daltonics, Billerica, Massachusetts). Bacterial isolates were determined to be susceptible or resistant (intermediate were classified as resistant) based on published Clinical and Laboratory Standards Institutes (CSLI) veterinary breakpoints using a dilution susceptibility technique.^29^ When breakpoints for cats were not available, susceptible or resistant classifications were extrapolated based on published breakpoints used in humans or dogs.^30^ For *Pasteurella* spp., breakpoints were extrapolated from Enterobacterales breakpoints. When none of the previous were available, data were excluded from antimicrobial resistance analysis. Isolates were further classified as multi‐drug resistant (MDR) based on the current definition from the Center for Disease Control, whereby a pathogen is considered MDR if it is resistant to 1 agent in ≥3 antimicrobial categories in which the wild type bacteria would normally be susceptible^31^ (Table S1).

### 2.2 Statistical methods

Data were assessed for normality using the Shapiro‐Wilk test. Data are presented as number and proportion for categorical data and for continuous data not normally distributed as median (interquartile range). The number of isolates noted in cats with UTI, SBU, and pyelonephritis was compared among categories using a Fisher's exact test. Signalment, recent administration antimicrobials, year of culture, comorbidities, and classification of UTI or SBU were evaluated using multivariable logistic regression model that was conducted in a backward stepwise manner to estimate the odds ratio (OR) of antimicrobial resistance for each of the 13 antimicrobials for which data was available and for multidrug resistance as the independent variable with and without a constant (Table S2). All models were assessed by comparing the expected and observed values using the Hosmer‐Lemeshow goodness of‐fit test. A *P*‐value <.05 was considered to represent an inadequate model fit and the model with the highest Nagelkerke *R* square value was chosen. All statistical analyses were performed using commercially available software (IBM SPSS Statistics for Macintosh, Version 28.0, IBM Corp, Armonk, New York).

## 3 RESULTS

Over the 8‐year study period, 559 positive ABUC were identified. A total of 363 positive ABUC from 308 cats met the inclusion criteria. Forty‐seven positive ABUC were excluded because the urine specimen was not collected by cystocentesis, SC ureteral bypass port or nephropyelocentesis. Forty‐four additional positive ABUC also were excluded because of low bacterial growth (<10^3^ cfu/mL) or suspected specimen contamination. Finally, 105 positive ABUC were excluded as because they represented subsequent positive ABUC from cats with multiple positive ABUC in a single calendar year.

Thirty‐nine cats had bacteriuric episodes in multiple calendar years. Of the 308 cats included with positive ABUC, 269 cats had a single positive ABUC, 27 appeared in 2 different calendar years, 8 appeared in 3 different calendar years and 4 appeared in 4 different calendar years. No cats appeared in >4 calendar years. At the time the positive ABUC was documented, the median age of the cats was 12 years (interquartile range [IQR], 7.5‐15 years) and the range was 6 months to 23 years. Thirty‐two (8.8%) cats were <5 years old, 93 (25.6%) were 5‐10 years old, 225 (62%) were >10 years old and for 13 (3.6%) cats the age was unknown. Eleven cats (3%) were female intact, 248 (68.3%) were female spayed, 7 (1.9%) were male intact, and 97 (26.7%) cats were male castrated. Seventeen breeds were represented including Domestic Shorthair 199 (44.1%), Domestic Longhair 60 (13.3%), Domestic Medium‐hair 49 (10.9%), Maine Coon 11 (2.4%), Siamese 10 (2.2%), and ≤7 (<2%) of each of the following: mixed breed, Manx, Bengal, Bobtail, Himalayan, Abyssinian, Birman, Burmese, Persian, Ragdoll, Somali, and American Shorthair.

Broadly categorized, 213 (58.7%) of the 363 positive ABUC specimens were from cats classified with SBU, 108 (29.8%) with sporadic bacterial cystitis, and 13 (3.6%) with recurrent bacterial cystitis (Table 1). For 29 (7.9%), a classification could not be determined. Three (1.4%) of the positive ABUC specimens classified as SBU also were classified as being recurrent. Across the classifications of SBU, recurrent bacterial cystitis and sporadic bacterial cystitis, 70 specimens were further classified as pyelonephritis, persistent or relapsing, or both. Pyelonephritis was the most common overlapping classification (n = 56 [15.4%]).

**TABLE 1:** Classification of 363 bacteriuric episodes from 308 cats over an 8‐year period.

Classification	Number of episodes
Subclinical bacteriuria	
Subclinical bacteriuria only	155 (42.7)
Pyelonephritis	40 (11.0)
Persistent or relapsing^a^	14 (3.9)
Recurrent^b^	3 (0.8)
Pyelonephritis and persistent or relapsing	1 (0.3)
Sporadic bacterial cystitis	
Sporadic bacterial cystitis only	94 (25.9)
Pyelonephritis	14 (3.9)
Recurrent bacterial cystitis	
Recurrent bacterial cystitis only	12 (3.3)
Pyelonephritis	1 (0.3)
Unknown classification	29 (7.9)

^a^ For the purposes of this study, persistent or relapsing was defined as a bacterial urinary tract infection that did not clear between urine cultures while a patient was actively receiving antimicrobials, or the term relapsing was used to describe the infection in the clinical diagnosis section of the medical record.

^b^ Despite not having overt lower urinary tract signs, 3 cats were classified as subclinical and recurrent because they had 3 or more positive urine cultures in the preceding 6 months.

Comorbidities were documented in 323/363 (89%) of the cats at the time of the bacteriuric episode (see Table 2). Different comorbidities could have been present in the same cat at different time points. One hundred sixty‐six (45.7%) cats had only 1 comorbidity and 157 (43.3%) had >1. Kidney disease (including both acute and chronic) was the most common comorbidity and was documented for 216 bacteriuric episodes (59.5% of all cats in the study and 66.9% of cats with comorbidities). Other common comorbidities included gastrointestinal disease, lower urinary tract disease, anemia and neoplasia. Two hundred ninety‐one (80.2%) cats at the time of their bacteriuric episodes were not receiving antimicrobials, 67 (18.5%) cats had current or recent antimicrobial administration, and history was not available for 5 (1.3%) cats.

**TABLE 2:** List of comorbidities from 363 bacteriuric episodes from 308 cats over an 8‐year period.

Disease^a^	Number of disease episodes
Kidney disease	219 (37.1)
Gastrointestinal disease^b^	71 (12)
Lower urinary tract disease^c^	46 (7.8)
Anemia	45 (7.6)
Neoplasia	44 (7.5)
Hyperthyroidism	32 (5.4)
Cardiovascular disease^d^	31 (5.3)
Neurologic disease	26 (4.4)
Pancreatitis	24 (4.1)
Diabetes mellitus	12 (2.0)
Hepatic disease	7 (1.2)
Miscellaneous Orthopedic disease, septic abdomen, oral disease, upper or lower airway disease, mycoplasma infection, dermatologic disease, gastric foreign body, chylothorax, pyometra, viral infections, and endocarditis	33 (5.6)

^a^ Total number of diseases exceeds the number of cats as many cats had multiple comorbidities.

^b^ Includes gastroenteritis, inflammatory bowel disease. Gastrointestinal neoplasia (eg, alimentary lymphoma) included under neoplasia.

^c^ Includes feline idiopathic cystitis, urolithiasis and anatomical abnormalities including previous perineal urethrostomy.

^d^ Includes cardiac disease (eg, hypertrophic cardiomyopathy, congestive heart failure) and systemic hypertension.

**FIGURE 1:**
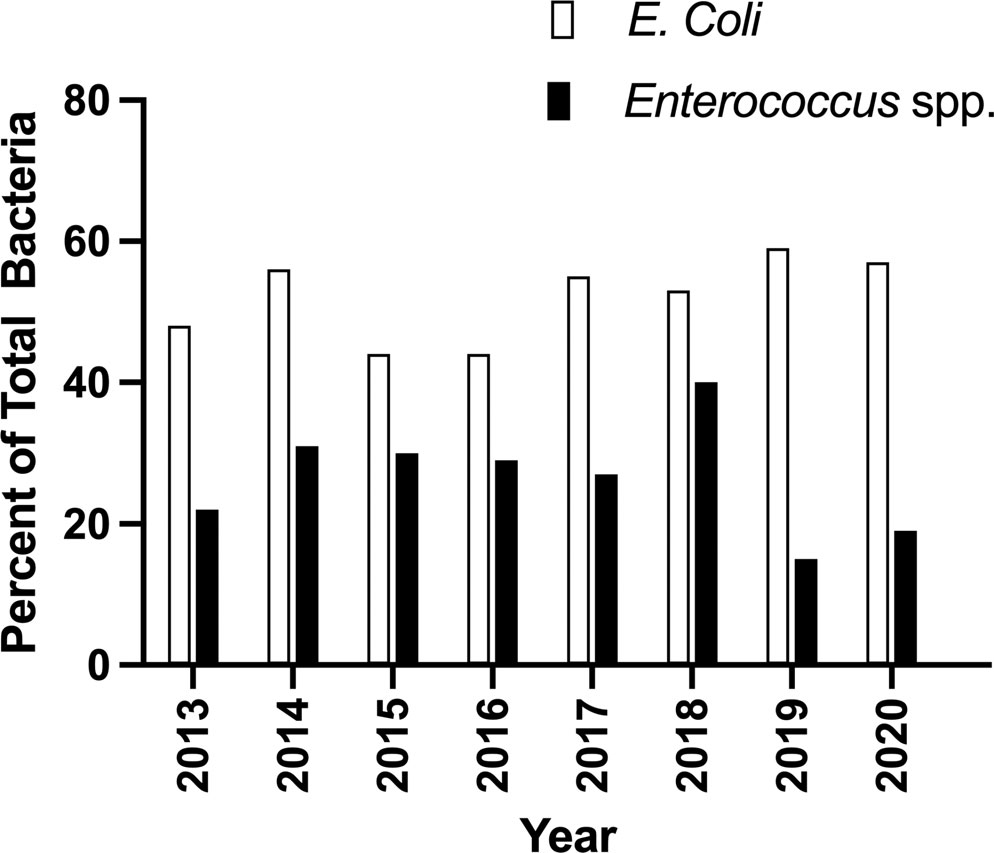
Percent isolates of *Escherichia coli* and *Enterococcus* spp. of total bacteria per calendar year obtained from 363 bacteriuric episodes in cats over an 8‐year period.

The majority of the positive ABUC (290 [79.9%]) were monomicrobial, whereas 73 (20.1%) were polymicrobial. From the 363 positive urine cultures, 444 specific bacterial isolates were identified, representing 31 species (Table 3). Two hundred sixty‐five (59.7%) isolates were gram‐negative and 179 (40.3%) were gram‐positive. *Escherichia coli* was the most common bacterial species isolated overall (231/444 [52.0%]). Of the gram‐negative organisms, 251 (95%) were enteric species and, of these, *E. coli* (hemolytic and non‐hemolytic) was most common (231 [92%]). The distribution of *E. coli* and *Enterococcus* spp. over time is presented in Figure 1.

**TABLE 3:** Bacterial species classified by type of bacteriuric episode from positive aerobic bacterial urine culture from cats.

Bacterial species	Total isolates, n (%)	Subclinical, n (%)	Sporadic bacterial cystitis, n (%)	Recurrent bacterial cystitis, n (%)	Pyelonephritis, n (%)	Unknown classification, n (%)
Total number of isolates	444	264	126	15	65	39
Gram‐negative						
Enteric						
*Escherichia coli*	231 (52.0)	115 (43.5)	89 (70.6)	10 (66.7)	36 (55.4)	17 (43.6)
*Enterobacter* spp.	8 (1.8)	7 (2.7)	1 (0.8)	‐	‐	‐
*Proteus mirabilis*	7 (1.6)	1 (0.4)	5 (4)	‐	‐	1 (2.6)
*Klebsiella* spp.	5 (1.1)	3 (1.1)	1 (0.8)	‐	2 (3.1)	1 (2.6)
Non‐enteric						
*Pseudomonas aeruginosa*	12 (2.7)	9 (3.4)	‐	1 (6.7)	5 (7.7)	2 (5.1)
Other non‐enteric species^a^	2 (0.5)	1 (0.4)	1 (0.8)	‐	‐	‐
Gram‐positive						
*Enterococcus faecalis*	90 (20.3)	64 (24.2)	17 (13.5)	2 (13.3)	12 (18.4)	7 (17.9)
*Enterococcus faecium*	17 (3.8)	13 (4.9)	‐	‐	4 (6.1)	4 (10.3)
Other *Enterococcus* spp.	10 (2.2)	8 (3)	1 (0.8)	1 (6.7)	1 (1.5)	‐
Coagulase negative *Staphylococcus*	26 (5.9)	18 (6.8)	7 (5.6)	‐	2 (3.1)	1 (2.6)
Other S*taphylococcus* spp.						
*Streptococcus* spp.	23 (5.2)	15 (5.7)	3 (2.4)	1 (6.7)	2 (3.1)	4 (10.3)
*Corynebacterium urealyticum*	12 (2.7)	9 (3.4)	1 (0.8)	‐	1 (1.5)	2 (5.1)
	1 (0.2)	1 (0.4)	‐	‐	‐	‐

^a^ *Actinobacter baumannii, Pasteurella dagmatis*.

*Enterococcus* spp. were more likely to be isolated from cats with SBU compared to those with sporadic or recurrent bacterial cystitis (32.2% vs 10.8%, *P* < .001). *Escherichia coli* was significantly less likely to be isolated from cats with SBU compared to those with other types of bacteriuria (43.6% vs 26.1%, *P* < .001), and significantly more likely to be isolated from cats with sporadic bacterial cystitis compared to those with recurrent cystitis or SBU (70.6% vs 32.0%, *P* < .001). Of the 56 ABUC that were subclassified as pyelonephritis, 49 were monomicrobial, and 7 were polymicrobial. Sixty‐five organisms were isolated with *E. coli* representing the majority (55.4% [36/65]) and *Enterococcus* spp. representing 26.2% (17/65). Seven other bacterial species were isolated from cats with pyelonephritis, but each represented <8% of the total isolates.

When evaluating the effect of time, positive ABUC that were evaluated in later calendar years represented a risk factor for resistance for multiple antimicrobials (OR ranging from 0.99 to 1.1) (Table 4). The most notable increase in resistance over time was to amoxicillin/clavulanic acid (OR, 1.1; 95% CI, 1.0‐1.2; p = .05). Isolates from cats with recurrent bacterial cystitis had significantly higher OR (3.9) and 95% CI (1.3‐11.3, *P* = .012) of being resistant to amoxicillin/clavulanic acid than isolates from other classifications of bacteriuria. Isolates from cats with both SBU and sporadic bacterial cystitis had significantly lower odds for resistance to doxycycline and being classified as MDR, compared to isolates from other classifications of bacteriuria. The presence of comorbidities and associations with antimicrobial resistance are presented in Table 4. Current or recent antimicrobial administration was associated with an increase OR for bacterial resistance only to erythromycin (OR, 2.5; 95% CI, 1.1‐6.1; *P* =  .04).

**TABLE 4:** Results of multivariate logistic regression analysis from 363 positive aerobic bacterial urine cultures in cats for each antimicrobials and presence of multidrug resistance.

	Year of submission	Recurrent bacterial cystitis	Subclinical UTI	Sporadic bacterial cystitis	Current or recent antimicrobial use	Kidney disease	GI disease	No comorbidity	Miscellaneous comorbidities
Amikacin 0.26	1.0 (1.0‐1.0) *0.014*	NS	NS	NS	NS	NS	NS	0.38 (0.15‐0.96) *0.04*	NS
Amoxicillin/Clavulanic acid 0.35	1.1 (1.0‐1.2) *0.05*	3.9 (1.3‐11.3) *0.012*	NS	NS	NS	NS	NS	NS	NS
Ampicillin 0.13	1.0 (1.0‐1.0) *<0.001*	NS	NS	NS	NS	NS	1.88 (1.1‐3.2) *0.021*	NS	NS
Chloramphenicol 0.68	0.99 (0.98‐0.99) *<0.001*	NS	NS	NS	NS	NS	NS	NS	NS
Doxycycline 0.44	NS	NS	0.41 (0.30‐0.56) *<0.001*	0.39 (0.25‐0.59) *<0.001*	NS	NS	NS	NS	NS
Enrofloxacin 0.53	0.99 (0.99‐1.0) *<0.001*	NS	NS	NS	NS	NS	NS	NS	NS
Erythromycin 0.34	NS	NS	NS	NS	2.5 (1.1‐6.1) *0.037*	NS	NS	NS	NS
Gentamicin 0.26	NS	NS	NS	NS	NS	0.43 (0.31‐0.60) *<0.001*		0.44 (0.21‐0.94) *0.035*	
Imipenem 0.67	0.99 (0.99‐0.99) *<0.001*	NS	NS	NS	NS	NS	NS	NS	NS
Marbofloxacin 0.48	0.99 (0.99‐1.0) *<0.001*	NS	NS	NS	NS	NS	NS	NS	NS
Nitrofurantoin 0.75	0.99 (0.99‐0.99) *<0.001*	NS	NS	NS	NS	NS	NS	NS	NS
Tetracycline 0.36	1.0 (0.99‐1.0) *<0.001*	NS	NS	NS	NS	NS	NS	NS	2.7 (1.0‐7.4) *0.04*
TMS 0.64	0.99 (0.98‐0.99) *<0.001*	NS	NS	NS	NS	NS	NS	NS	NS
Multidrug resistance 0.46	NS	NS	0.19 (0.13‐0.28) *<0.001*	0.30 (0.19‐0.47) *<0.001*	NS	NS	NS	NS	0.27 (0.11‐0.64) *0.003*

*Note*: Data presented are odds ratio of resistance and (95 confidence interval) with *P*‐value. The value under the antimicrobial represents the Nagelkerke's pseudo *R*‐squared from the model. Variables entered into regression included signalment, year of culture, classification of urinary tract infection, comorbidities, and recent or current antimicrobial use. Miscellaneous comorbidities including orthopedic disease, viral infections, and airway disease among several others. Variables entered that were not significant (NS) in any model do not appear in this table.

Antimicrobial susceptibility results for all bacterial species isolated >10 times are shown in Table 5. Antimicrobial susceptibilities varied depending on both the bacteria and antimicrobial being tested. When evaluating all bacterial isolates, no antimicrobial was categorized as effective against >90% of isolates, and only chloramphenicol and imipenem achieved >80% effective. Nineteen percent of all bacterial isolates were considered MDR with a range of 8%‐56% in an individual species.

**TABLE 5:** Percent susceptibility of isolates to various antimicrobials when data was available for 10 or more bacterial isolates obtained from positive aerobic bacterial urine cultures obtained from cats over an 8‐year period.

	Isolate number	Amik	A/C	Ampi	Cefaz	Chlor	Clind	Doxy	Enro	Eryth	Gent	Imip	Marbo	Mino	Nitro	Oxa	P/T	Prado	Tetra	T/S	Vanco	MDR
All bacteria	444	72	72	62	49	86	‐	71	61	‐	71	88	59	‐	‐	‐	‐	41	39	75	‐	19
Enterobacterales	250	98	68	57	69	92	‐	86	93	‐	95	100	94	100	67	‐	97	95	86	96	‐	13
S*taphylococcus* spp.	48	96	67	42	73	81	71	79	79	67	85	71	53	72	100	73	‐	69	34	70	95	29
*E. coli* (hemolytic)	183	97	77	66	73	94	‐	92	99	‐	98	99	99	100	‐	‐	98	100	91	99	‐	8
*E. coli* (non‐hemolytic)	45	100	36	24	69	91	‐	78	76	‐	89	100	76	‐	‐	‐	100	83	79	89	‐	26
*Enterococcus faecalis*	90	‐	100	99	‐	83	‐	46	‐	40	‐	84	‐	43	‐	‐	‐	‐	40	80	98	24
*Enterococcus faecium*	17	‐	24	2	‐	94	‐	41	‐	24	‐	11	‐	44	‐	‐	‐	‐	11	38	100	56
*Pseudomonas aeruginosa*	12	100	‐	‐	‐	‐	‐	‐	60	‐	92	73	82	‐	‐	‐	92	25	‐	‐	‐	31

*Note*: Multidrug resistance is reported as percent of isolates that were defined according to CDC guidelines.^29^

Abbreviations: A/C, Amoxicillin/Clavulanic acid; Amik, Amikacin; Ampi, Ampicillin; Cefaz, Cefazolin; Chlor, Chloramphenicol; Clind, Clindamycin; Doxy, Doxycycline; Eryth, Erythromycin; Gent, Gentamicin; Imip, Imipenem; Marbo, Marbofloxacin; MDR, multidrug resistance; Nitro, Nitrofurantoin; Oxa, Oxacillin; P/T, Pipercillin/Tazobactam; Prado, Pradofloxacin; T/S, Trimethoprim/Sulfamethoxazole; Tetra, Tetracycline; Vanco, Vancomycin.

## 4 DISCUSSION

A clinically relevant increase in antimicrobial resistance was noted over time for only 1 antimicrobial (amoxicillin/clavulanic acid) despite the prevalence of bacterial organisms remaining static during the study period. Although no risk factors or trend changes for MDR isolates were documented, 19% of all bacterial isolates were classified as MDR. The time frame we investigated represented a uniform period of antimicrobial susceptibility testing protocols to include the most recent CLSI veterinary breakpoints where applicable. The inclusion criteria of only 1 positive ABUC per cat per calendar year was used to minimize bias when trying to evaluate resistance over time because several cats had numerous positive ABUC each year.

In our study, the median age of cats at the time of the bacteriuric episode was 12 years and the majority of cats (62%) were >10 years of age. In other studies, the median ages were 8 and 10 years^13^, ^23^ and the mean ages were 5.7 and 10.9 years.^4^, ^27^ However, in another study evaluating only SBU, the median age was 14 years.^17^ The difference in age at presentation may be related to the presence of comorbidities, clinician discretion in submitting a urine culture and variable methods for urine specimen collection, with studies in which median or mean age was younger including voided and catheterized specimens. Furthermore, geographic location, inclusion criteria such as the presence or absence of clinical signs, and patient characteristics of cats seen at primary as compared to tertiary care hospitals also might have contributed to these differences.

The majority (89%) of cats in our study had at least 1 comorbidity. Kidney disease was the most common comorbidity followed by gastrointestinal disease, lower urinary tract disease, anemia and neoplasia. Numerous studies over the past 15 years have evaluated comorbidities as potential risk factors for UTI and SBU in cats, but control groups often were missing. With the exception of anemia, all comorbidities identified in our study have been reported in other studies.^12^, ^13^, ^14^, ^15^, ^27^ The presence of anemia may have been associated with the other diseases previously mentioned, or simply more urine specimens may have been submitted from anemic cats at our institution during this time period. We aimed to identify if comorbidities were risk factors for antimicrobial resistance; no control group was analyzed to determine if any of the comorbidities were risk factors for sporadic bacterial cystitis or SBU.

Subclinical bacteriuria was the most common classification of the bacteriuric episodes (58.7%) compared to cats with sporadic or recurrent classifications. This finding is in contrast to 2 studies of cats with positive urine cultures in which SBU was present in 35% and 40% of cats.^13^, ^27^ Only 1 other study had a higher percentage of SBU in its population of cats (75%).^18^ Comparisons among these studies are difficult to make because of variable inclusion and exclusion criteria. We did not evaluate temporal trends of bacteriuria classification in our study, but it is possible that in the later years of our study fewer ABUC were performed empirically because of increasing awareness of non‐treatment of SBU. This possibility could have decreased the proportion of SBU diagnosed and increased the likelihood of other classifications. We did not compare the bacterial species isolated from cats with pyelonephritis to those without pyelonephritis. The majority of cats with pyelonephritis were primarily classified as SBU (73%), the most common bacteria isolated from these ABUC was *E. coli*, not *Enterococcus*. This finding is despite *Enterococcus* species being more likely to be isolated from cats with SBU compared to cats with sporadic bacterial cystitis. In general, classification of bacteriuria is important because treatment for SBU with antimicrobials often is not indicated.^21^, ^32^ Treatment and outcome for cats with bacterial cystitis or SBU was beyond the scope of our study.

The most common bacterial species in our population of cats was *E. coli*, followed by *Enterococcus* (*faecalis* and *faecium*), *Staphylococcus* spp. (coagulase negative and other species), *Streptococcus* spp. and *Pseudomonas aeruginosa*. These findings are in agreement with most previous reports with all but 2 studies citing *E. coli* as the most common bacterial species isolated from cats.^16^, ^17^ Comparing bacterial species across the different classifications of bacteriuria, *Enterococcus* spp. was significantly more likely to be isolated from cats with SBU compared to other types of bacteriuric episodes. Similar findings were noted in other studies although no correlation between *Enterococcus* and SBU was found in 1 study.^14^, ^23^, ^27^ *Enterococcus* spp. has been isolated from the urine of healthy people, dogs and cats, and frequently is considered a commensal organism of the gastrointestinal tract with low virulence compared to other pathogens such as *E. coli*.^33^ In dogs, risk factors found to be associated with *Enterococcus* UTIs included urolithiasis, lower urinary tract neoplasia and a history of recurrent infection.^34^ These findings add to the body of evidence that *Enterococcus* spp. may be associated with SBU and not represent true UTI. *Escherichia coli* was significantly more likely to be isolated from cats with sporadic bacterial cystitis than cats with recurrent bacterial cystitis, SBU or pyelonephritis. In 2 studies, *E. coli* was the most common bacterial species isolated from cats with LUTS.^23^, ^27^ Not only is *E. coli* the most common cause of UTI in dogs and cats, but some species of *E. coli* have a number of virulence factors that might lead to inflammation and clinical signs of UTI.

Over the study period, a significant and clinically relevant increase in the odds (OR, 3.9; 95% CI, 1.3‐11.3) of antimicrobial resistance to amoxicillin/clavulanic acid was observed over time. Although many studies have investigated overall percentages of antimicrobial resistance in companion animals with UTI, few have determined whether or not a temporal change has occurred. An increase in antimicrobial resistance of urinary isolates from cats has been noted in some studies, but not in others. Two studies,^23^, ^27^ found no change in resistance to amoxicillin/clavulanic acid over the time periods evaluated (2009‐2014 and 2000‐2009, respectively), whereas another study reported an increase in antimicrobial resistance when evaluating isolates from 1999 to 2014.^35^ The overall percentage susceptibility of isolates to amoxicillin/clavulanic acid in our study appears less than that found in several older studies,^5^, ^12^, ^36^ but comparisons must be made with caution because CLSI breakpoints for many antimicrobials have decreased with the publication of each new set of guidelines and laboratory implementation of urine versus serum breakpoints may vary. Despite International Society of Companion Animal Infectious Disease (ISCAID) guidelines not recommending amoxicillin/clavulanic acid as initial treatment for sporadic bacterial cystitis, veterinarians often still prescribe this drug in the United States and Canada for cats with UTI.^37^ One study noted that amoxicillin/clavulanic acid was prescribed with increasing frequency over a 3‐year period (2016‐2018).^37^ In 2 other studies investigating antimicrobial administration to cats and dogs, amoxicillin/clavulanic acid was consistently the most commonly prescribed antimicrobial.^26^, ^38^ Another study evaluating antimicrobial administration to dogs from 2016 to 2018 documented that amoxicillin/clavulanic acid was still the most frequently prescribed antimicrobial if bacterial cystitis was suspected, but an overall increase in first line antimicrobials such as amoxicillin and trimethoprim/sulfamethoxazole also was noted during that time.^39^ This observation is similar to what has been reported in people, and studies in both adults and pediatric patients have shown an increase in resistance to amoxicillin/clavulanic acid, particularly for *E. coli*.^40^, ^41^ An increase in antimicrobial resistance to amoxicillin/clavulanic acid also was seen in cats with recurrent bacterial cystitis compared to sporadic bacterial cystitis and SBU. The reasons for this finding are not well understood because current or recent antimicrobial administration was not associated with an increase in resistance to amoxicillin/clavulanic acid in our study. This finding might be a consequence of the retrospective nature of the study, and not all medical records adequately documented previous use of antimicrobials. Prior antimicrobial administration was only found to be associated with an increase in resistance to erythromycin. The reason for this finding is unclear and likely represents a statistical association without causation.

Cats with gastrointestinal disease had a significant increase in resistance to ampicillin. Cats with chronic enteropathies (e.g., inflammatory bowel disease, alimentary lymphoma) have been reported to have an altered intestinal microbiome with decreased numbers of beneficial bacteria and increased numbers of problematic bacteria such as *E. coli* and *Streptococcus*.^42^, ^43^ In a study comparing the fecal microbiota of healthy cats and cats with chronic enteropathies, an increase in the dysbiosis index was found in 76% of cats with chronic enteropathies.^44^ In people, an altered intestinal microbiome has been reported to be a reservoir for MDR bacteria, particularly in people hospitalized for chronic enteropathies.^45^ Although evidence is lacking, it is reasonable to consider an association between dysbiosis and bacteriuria in cats with chronic enteropathies and further prospective studies are warranted. We also noted that cats with kidney disease had a significantly lower risk for resistance to gentamicin. Although in theory this observation could be attributed to the fact that gentamicin, a potentially nephrotoxic antimicrobial, is rarely administered to cats with kidney disease, it is also true that gentamicin is rarely administered to any patients at our institution. Furthermore, cats with no comorbidities in our study also had a lower risk of resistance to gentamicin.

No antimicrobial tested achieved a >90% effectiveness designation for all bacteria isolated. This finding is similar to most studies of antimicrobial susceptibility of bacterial urinary tract pathogens in cats.^28^, ^46^, ^47^, ^48^ The percentage susceptibility of all bacterial isolates to amoxicillin/clavulanic acid, trimethoprim/sulfamethoxazole and enrofloxacin, 3 commonly prescribed antimicrobials, was similar to what was previously reported in cats from Norway, but differed from a study evaluating urinary isolates from cats in Taiwan where all isolates had significantly lower susceptibility to these same antimicrobials.^28^, ^49^ Geographical differences and different antimicrobial prescribing patterns are likely reasons for the variation in results. Our findings support the ISCAID guidelines regarding empirical use of amoxicillin or trimethoprim/sulfamethoxazole pending urine culture results, if clinically indicated, because >60% of the bacteria were susceptible to these antimicrobials, yet highlights the need for culture and susceptibility testing. The antimicrobials for which all bacterial isolates had the highest susceptibility in our study were chloramphenicol and imipenem. This finding is not unexpected because these antimicrobials typically are not used as first line treatments of UTI or other infections and generally are reserved to treat MDR or life‐threatening infections.

*Escherichia coli* had the lowest susceptibility to ampicillin (66%), cefazolin (73%), and amoxicillin/clavulanic acid (77%), but *E. coli* rarely was documented to have MDR (8%). *Escherichia coli* isolates in our study had higher overall susceptibility to the antimicrobials tested compared to other studies,^25^, ^28^, ^47^, ^49^ particularly when comparing ampicillin and amoxicillin/clavulanic acid. However, other studies reported slightly higher susceptibility to amoxicillin/clavulanic acid.^46^, ^48^ At our institution, we frequently submit ABUC and susceptibility testing for cats with suspected UTI versus starting empirical treatment with antimicrobials, which might help decrease inappropriate antimicrobial administration and potential resistance. Thus, geographical differences for antimicrobial prescribing patterns are likely the reason for the different susceptibilities found in the various studies.

In our study, the majority of *Enterococcus* isolates were *E. faecalis*. This distribution is similar to infections in dogs and humans.^33^, ^34^ *Enterococcus faecium* has higher intrinsic resistance to penicillins and overall MDR classification, which was noted in our study. *Enterococcus faecalis* was found to have a 24% incidence of MDR status whereas 56% of *E. faecium* were classified as MDR. Other studies have noted lower rates of MDR *Enterococcal* infections, but these included not only urinary isolates, but isolates obtained from other sources.^24^, ^50^, ^51^ The comorbidities present in our cats may have biased some clinicians to submit urine for culture when clinical signs were not present, particularly in the early years of the study period. Given the high percentage of SBU in our study and its association with *Enterococcus* spp., a higher percentage of MDR *Enterococcus* spp. may have been identified in our study.

*Staphylococcus pseudintermedius* had the highest percentage of MDR compared to all other isolates we evaluated. This finding is similar to a study of dogs and cats from Canada, but this study included isolates obtained from other sources, in addition to urine.^51^ A temporal increase in MDR *S. pseudintermedius* ^35^ was reported over 16 years in a study evaluating urinary tract bacterial isolates from dogs and cats. *Staphylococcus pseudintermedius* is the most common *Staphylococcus* species and it has developed antimicrobial resistance leading to widespread methicillin‐resistant *Staphylococcus pseudintermedius* infections in humans and animals.^52^ Many infections remain susceptible only to second‐ and third‐line antimicrobials such as aminoglycosides and chloramphenicol, and growing concern exists about pan‐drug resistance, particularly as crossover infections between humans and animals become more common.^53^ Despite a slight increase in antimicrobial resistance to some antimicrobials over the study period, it was not substantial enough to result in a corresponding significant increase in multidrug resistance. Overall, limited information is available describing the prevalence of MDR infections in bacteriuria from cats. Thus, more studies specifically evaluating antimicrobial resistance are needed.

Our study had several limitations inherent to retrospective studies. We relied on the pertinent history in the medical record for information such as the presence or absence of LUTS, prior antimicrobial use, and description of comorbidities. In several cases, this information was missing or unclear, and thus could have affected how cats were categorized. Moreover, even when complete documentation was available in the medical record about the presence or absence of LUTS, some signs may have gone unwitnessed by the owner. In a study that compared owner reported litter box elimination behavior versus a video recording system (VRS), the number of urination events as documented by the VRS was significantly higher than what owners reported.^54^ In addition, we relied on the attending clinician's assessment for the clinical diagnosis, particularly in the case of pyelonephritis. Therefore, it is possible that cats could have been misclassified regarding their type of bacteriuric classification. Furthermore, a small number of cats in our study had concurrent or recent urethral obstruction. Thus, these cats could have been classified incorrectly as having bacterial cystitis, when their clinical signs in fact were a consequence of their obstructive lower urinary tract disease. Because we only included cats with a positive ABUC, the true prevalence of bacterial cystitis or SBU could not be determined. Our institution is a large tertiary referral and emergency hospital, which might bias our population of cats, including more cats with SBU and recurrent UTIs, compared to sporadic bacterial cystitis. However, we only included 1 positive urine culture per cat per calendar year to minimize bias for recurrent or persistent UTI. Cats that had follow‐up and positive urine cultures at another veterinary facility also may have been misclassified, potentially affecting the results. A final limitation is that some samples had multiple bacteria isolated from the same sample, which can increase the likelihood of type 1 statistical error in the logistic regression analysis of antimicrobial resistance.

## 5 CONCLUSION

No significant change in the prevalence of bacterial species was evident during the study period, but a significant and clinically relevant increase in antimicrobial resistance to amoxicillin/clavulanic acid was found. Prior or current antimicrobial use did not increase the risk for antimicrobial resistance to most antimicrobials commonly prescribed for UTI. No antimicrobial tested achieved >90% effectiveness for all bacteria isolated and MDR was common. Data from our retrospective study illustrates that no reliable gold standard antimicrobial choice is available for treating UTIs in cats, and resistance to commonly prescribed antimicrobials is increasing. Urine culture and susceptibility testing in cats with LUTS is warranted, particularly when clinical signs are recurrent.

## CONFLICT OF INTEREST DECLARATION

Authors declare no conflict of interest.

## OFF‐LABEL ANTIMICROBIAL DECLARATION

Authors declare no off‐label use of antimicrobials.

## INSTITUTIONAL ANIMAL CARE AND USE COMMITTEE (IACUC) OR OTHER APPROVAL DECLARATION

Authors declare no IACUC or other approval was needed.

## HUMAN ETHICS APPROVAL DECLARATION

Authors declare human ethics approval was not needed for this study.

## Supporting information


**Table S1:** List of antimicrobial agents evaluated for various bacterial genus to determine if multidrug resistance was present.
**Table S2:** List of dependent factors that were entered into logistic regression analysis for each antimicrobial assessed and multidrug resistance of a bacterial pathogen.
