# Supplementary material for: Antimicrobial susceptibility patterns from urinary isolates obtained from cats (2013‐2020)
Source: J Vet Intern Med. 2023 Apr 19;37(3):1077–87. doi: 10.1111/jvim.16711 (PMC10229343; doi:10.1111/jvim.16711)
Supplement: Supplementary file 1 — Table S1: List of antimicrobial agents evaluated for various bacterial genus to determine if multidrug resistance was present. Table S2: List of dependent factors that were entered into logistic regression analysis for each antimicrobial assessed and multidrug resistance of a bacterial pathogen. [file JVIM-37-1077-s001.pdf]

Supplemental Table 1: List of antimicrobial agents evaluated for various bacterial genus to determine if multidrug resistance was present.

***Staphylococcus* species**

Antimicrobial category	Agent(s)
Aminoglycoside	Amikacin<br>Gentamicin
Ansamycin	Rifampin
ß-lactams	Oxacillin
Fluoroquinolone	Enrofloxacin<br>Marbofloxacin
Folate pathway inhibitor	Trimethoprim/Sulphamethoxazole
Glycopeptide	Vancomycin
Lincosamide	Clindamycin
Macrolide	Erythromycin
Nitrofuran	Nitrofurantoin
Phenicol	Chloramphenicol
Tetracyclines	Doxycycline<br>Minocycline<br>Tetracycline

***Enterococcus* Species**

Antimicrobial category	Agent
Penicillins	Ampicillin<br>Amoxicillin/Clavulanate
Carbapenem (not E. faecium)	Imipenem
Glycopeptide	Vancomycin
Phenicol	Chloramphenicol
Nitrofuran	Nitrofurantoin
Tetracyclines	Doxycycline<br>Minocycline<br>Tetracycline

Enterobacterales family

Antimicrobial category	Agent
Aminoglycoside	Amikacin<br>Gentamicin
Anti-pseudomonal penicillin	Piperacillin/Tazobactam
Carbapenem	Imipenem
1st generation cephalosporin	Cefazolin
3rd generation cephalosporin	Ceftazidime
Fluoroquinolone	Enrofloxacin<br>Marbofloxacin
Folate pathway inhibitor	Trimethoprim/Sulphamethoxazole
Penicillin	Ampicillin
Penicillin + ß-lactamase inhibitor	Amoxicillin/clavulanate
Phenicol	Chloramphenicol
Nitrofuran	Nitrofurantoin
Tetracycline	Doxycycline<br>Minocycline<br>Tetracycline

Pseudomonas aeruginosa* or *Acinetobacter baumannii

Antimicrobial category	Agent
Aminoglycoside	Amikacin<br>Gentamicin
Anti-pseudomonal penicillin	Piperacillin/Tazobactam
Carbapenem	Imipenem
3rd generation cephalosporin	Ceftazidime
Fluoroquinolone	Enrofloxacin

Pasteurella spp.

Antimicrobial category	Agent
Aminoglycoside	Amikacin<br>Gentamicin
Anti-pseudomonal penicillin	Piperacillin/Tazobactam
Carbapenem	Imipenem
1st generation cephalosporin	Cefazolin
3rd generation cephalosporin	Ceftazidime
Fluoroquinolone	Enrofloxacin<br>Marbofloxacin
Folate pathway inhibitor	Trimethoprim/Sulphamethoxazole
Penicillin	Ampicillin
Penicillin + ß-lactamase inhibitor	Amoxicillin/clavulanate
Phenicol	Chloramphenicol
Tetracycline	Doxycycline<br>Tetracycline

Proteus mirabilis

Antimicrobial category	Agent
Aminoglycoside	Amikacin<br>Gentamicin
Anti-pseudomonal penicillin	Piperacillin/Tazobactam
Carbapenem	Imipenem
1st generation cephalosporin	Cefazolin
3rd generation cephalosporin	Ceftazidime
Fluoroquinolone	Enrofloxacin<br>Marbofloxacin
Folate pathway inhibitor	Trimethoprim/Sulphamethoxazole
Phenicol	Chloramphenicol

Supplemental Table 2: List of dependent factors that were entered into logistic regression analysis for each antimicrobial assessed and multidrug resistance of a bacterial pathogen.

Sex
Age
Current or recent antimicrobial administration
Classification of bacteriuria
- Subclinical
- Sporadic bacterial cystitis
- Recurrent bacterial cystitis
- Pyelonephritis
- Persistent or Relapsed urinary tract infection
- Unknown

Comorbidities
- Lower urinary tract abnormalities
- Gastrointestinal disease
- Pancreatitis
- Cardiovascular disease
- Hyperthyroidism
- Neoplasia
- Anemia
- Diabetes Mellitus
- Hepatic disease
- Neurologic disease
- Other
